# Automated Identification of Different Severity Levels of Diabetic Retinopathy Using a Handheld Fundus Camera and Single-Image Protocol

**DOI:** 10.1016/j.xops.2024.100481

**Published:** 2024-02-07

**Authors:** Fernando K. Malerbi, Luis Filipe Nakayama, Gustavo Barreto Melo, José A. Stuchi, Diego Lencione, Paulo V. Prado, Lucas Z. Ribeiro, Sergio A. Dib, Caio V. Regatieri

**Affiliations:** 1 Federal University of São Paulo, Sao Paulo, Brazil; 2 Phelcom Technologies, Sao Carlos, Brazil

**Keywords:** Artificial intelligence, Diabetes, Handheld camera, Portable, Retina

## Abstract

**Purpose:**

To evaluate the performance of artificial intelligence (AI) systems embedded in a mobile, handheld retinal camera, with a single retinal image protocol, in detecting both diabetic retinopathy (DR) and more-than-mild diabetic retinopathy (mtmDR).

**Design:**

Multicenter cross-sectional diagnostic study, conducted at 3 diabetes care and eye care facilities.

**Participants:**

A total of 327 individuals with diabetes mellitus (type 1 or type 2) underwent a retinal imaging protocol enabling expert reading and automated analysis.

**Methods:**

Participants underwent fundus photographs using a portable retinal camera (Phelcom Eyer). The captured images were automatically analyzed by deep learning algorithms retinal alteration score (RAS) and diabetic retinopathy alteration score (DRAS), consisting of convolutional neural networks trained on EyePACS data sets and fine-tuned using data sets of portable device fundus images. The ground truth was the classification of DR corresponding to adjudicated expert reading, performed by 3 certified ophthalmologists.

**Main Outcome Measures:**

Primary outcome measures included the sensitivity and specificity of the AI system in detecting DR and/or mtmDR using a single-field, macula-centered fundus photograph for each eye, compared with a rigorous clinical reference standard comprising the reading center grading of 2-field imaging protocol using the International Classification of Diabetic Retinopathy severity scale.

**Results:**

Of 327 analyzed patients (mean age, 57.0 ± 16.8 years; mean diabetes duration, 16.3 ± 9.7 years), 307 completed the study protocol. Sensitivity and specificity of the AI system were high in detecting any DR with DRAS (sensitivity, 90.48% [95% confidence interval (CI), 84.99%–94.46%]; specificity, 90.65% [95% CI, 84.54%–94.93%]) and mtmDR with the combination of RAS and DRAS (sensitivity, 90.23% [95% CI, 83.87%–94.69%]; specificity, 85.06% [95% CI, 78.88%–90.00%]). The area under the receiver operating characteristic curve was 0.95 for any DR and 0.89 for mtmDR.

**Conclusions:**

This study showed a high accuracy for the detection of DR in different levels of severity with a single retinal photo per eye in an all-in-one solution, composed of a portable retinal camera powered by AI. Such a strategy holds great potential for increasing coverage rates of screening programs, contributing to prevention of avoidable blindness.

**Financial Disclosure(s):**

F.K.M. is a medical consultant for Phelcom Technologies. J.A.S. is Chief Executive Officer and proprietary of Phelcom Technologies. D.L. is Chief Technology Officer and proprietary of Phelcom Technologies. P.V.P. is an employee at Phelcom Technologies.

Diabetic retinopathy (DR) meets the criteria for a disease that warrants screening because of its asymptomatic phase before vision loss, its significance as a public health problem, the availability of tests, the existence of established and effective treatment if performed in a timely manner, and improved prognosis with early intervention.^1^^,^^2^ However, despite DR being one of the leading causes of vision loss among adults, having affected > 100 million adults worldwide in 2020, low rates of patients with diabetes undergo the recommended annual DR screening, even in developed countries.^2^^,^^3^ Among the causes for such low adherence, socioeconomic and geographic barriers have been identified, as well as delayed referrals from primary care practitioners and inadequate patient education.^4^

Recent modalities, such as telemedicine, artificial intelligence (AI) systems, and portable retinal cameras, have been proposed to increase the uptake rates of DR screening.^5^ Among these, handheld retinal cameras are interesting tools for such tasks, because of their affordability and portability, enabling a wider coverage of screening programs. These devices can be utilized by primary care physicians or nonmedical personnel after proper training.^6^

The role of telemedicine in DR screening is continually expanding; the imaging protocol for DR evaluation has undergone a substantial evolution, from the 7-field ETDRS protocol to the more recent 2-field protocol, currently used for expert reading in many established programs.^7^ However, some barriers for wide-scale implementation of telemedicine DR screening are still present; some examples are the lack of adherence and operational challenges such as poor network access in rural areas.^8^^,^^9^ Hence, further work is necessary to continually optimize practices and improve long-term patient outcomes. In that sense, simplifying the imaging protocol holds the potential to increase adherence and improve the effectiveness of screening programs.

Recently, deep learning (DL) algorithms have emerged as a promising tool for the detection of DR, offering diagnostic performance comparable to human experts and the potential to scale up screening programs efficiently.^10^^,^^11^ The first such algorithm that received United States Food and Drug Administration (FDA) approval detects more-than-mild DR (mtmDR) because, from an ophthalmic perspective, only patients with mtmDR are currently recommended to receive specialized evaluation and consideration for treatment.^10^ However, the presence of DR is consistently associated with other complications of diabetes^12^; thus, the detection of any DR may also be part of a strategy to stratify the systemic risk of patients with diabetes. In that sense, DR screening strategies could be tailored to local circumstances, depending on the desired outcome and several additional factors, such as local epidemiology, available infrastructure, and workforce, allowing for effective implementation.

The aim of this study was to evaluate the performance of AI systems for the detection of both DR and mtmDR on fundus images obtained with a mobile, handheld retinal camera, utilizing a single retinal image protocol.

## Methods

### Study Design and Population

This was an observational cross-sectional study, in compliance with the tenets of the Declaration of Helsinki. The algorithmic performance was validated using a prospectively collected data set of retinal images from 327 individuals with diabetes enrolled at 3 different centers. One center is a tertiary referral ophthalmological hospital located in São Paulo, Brazil, whereas the other 2 are diabetes centers in São Paulo and Sergipe, Brazil, where DR evaluation is regularly performed. Informed consent was obtained from each patient before the study enrollment. The study was approved by the Research Ethics Committee of the Federal University of São Paulo (33.842.220.7.0000.5505). Inclusion criteria were patients with type 1 or 2 diabetes mellitus who agreed with the study terms. Exclusion criteria were a history of any other ocular disease that could impair DR classification, such as other maculopathies, uveitis, congenital malformations, media opacities that precluded retinal images of enough quality, and poor patient collaboration for the imaging protocol.

### Smartphone-Based Retinal Camera

The Eyer (Phelcom Technologies, LLC) is an FDA-approved smartphone-based camera built using a Samsung Galaxy S10 (Android 13) smartphone. The camera captures retinal fundus photographs with a 45° field angle. With a 12-megapixel sensor, it delivers an image at 1600 × 1600 pixels. It has an autofocus range from −20 to +20 diopters.

### Image Acquisition

All participants underwent pharmacological mydriasis through the administration of 2 drops of 0.5% tropicamide at 5-minute intervals, followed by image acquisition, consisting of 2 fundus images per eye, one macula centered and the other one optic disc centered. Images were acquired by previously trained health care professionals who already had experience in ocular imaging, using a standardized protocol.^13^ All images were anonymized, deidentified, and reviewed to ensure the removal of any personal health information.

### Grading Protocol

The ground truth was DR classification according to expert reading. Labeling was performed independently by 2 masked, certified ophthalmologists, with a third senior retinal specialist adjudicating in discordant cases. Classification of DR severity was performed according to the International Classification of Diabetic Retinopathy, based on 2 images per eye after mydriasis.^14^ Images were deemed gradable for human readers if ≥ 80% of the image area was visible and if the assessment of at least the third retinal vascular branch was possible.^15^

### Automated Detection of DR

Although the ground truth relied on expert reading using a 2-image protocol, we intended to assess the performance of automated detection of DR using a single image per eye. In order to achieve this goal, we employed 2 different DL systems, along with a combination of both, for the analysis of different severity levels of DR, after automated image quality evaluation. Such quality assessment was performed by a convolutional neural network (CNN), which was previously trained, based on factors including blurriness, exposure, presence of artifacts, media opacities, or incorrect fields of view.^16^ Images classified as gradable after the automatic quality evaluation underwent further automatic DR detection by 2 different DL systems, whose specific details are outlined below.

The first DL system utilized in this study for the detection of DR is the retinal alteration score (RAS), which has been designed and previously validated for the detection of retinal changes, and is described elsewhere.^17^ In essence, RAS is a modified version of the CNN Xception. It was initially trained using transfer learning on the EyePACS data set and then fine-tuned using a data set of 10,569 fundus images captured with the Phelcom Eyer device (resolution 1600 × 1600 × 3 red, green and blue channels). Images from the EyePACS data set had similar magnification and field of view as images obtained with the Eyer device. The training data were separated into 2 classes: images from normal eyes and images with retinal alterations.

Another DL-based approach employed in this study is the diabetic retinopathy alteration score (DRAS). It utilized a modified version of the EfficientNetV2S CNN, with different input and output parameters while maintaining the same intermediate convolutional layers. The input was modified to receive images of size 599 × 599 × 3 red, green and blue channels. Images were resized to reduce data dimensionality and also to enable faster convergence during the training of the network, in the best possible trade-off regarding the final performance. Additionally, the last 3 layers were dropped, and new layers of convolutional, batch normalization, activation, global average pooling, dense, and output layers were added. The network’s output represents the probability of alteration for the DR and non-DR classes. Therefore, the class to which the evaluated image belongs is identified based on the neuron with the highest value. DRAS gives an output as a numerical score (Table S1, available at www.ophthalmologyscience.org) ranging from 0 (low probability of DR) to 1 (high probability of DR).

The DRAS network went through a training process in which each image was individually evaluated, allowing its internal parameters to be progressively adjusted to obtain an output from the last layer that closely aligns with the corresponding image class. To train the algorithm, transfer learning was employed using the EyePACS data set along with an internal Phelcom data set comprising 17,330 DR images captured exclusively using the Eyer device (resolution 1600 × 1600 × 3 red, green and blue channels). For validation purposes, 30% of these images were used to periodically evaluate the performance of the network. To add more diversity, data augmentation was applied to images, with rotation, width and height shift, zoom, and brightness values randomly applied. This system’s output was assessed for classifying patients regarding the presence of DR.

**Figure 1:**
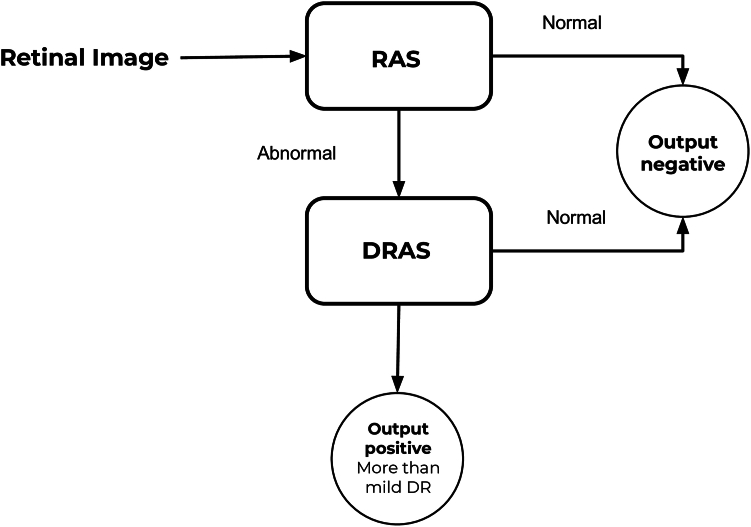
Artificial intelligence algorithms combination for detection of more-than-mild diabetic retinopathy (DR). DRAS = diabetic retinopathy alteration score; RAS = retinal alteration score.

Finally, images with a positive output from the RAS system were further analyzed by DRAS to provide the mtmDR output. (Fig 1) The combined networks (RAS and DRAS) analysis gives an output as a numerical score (Table S2, available at www.ophthalmologyscience.org) ranging from 0 (low probability of mtmDR) to 1 (high probability of mtmDR).

The implementation of the RAS and DRAS models is based on default Xception and EfficientNet v2s CNNs, respectively (Appendix S1, available at www.ophthalmologyscience.org). The only differences created by our group are that the input and output layers have been modified, so the input consists of resized images (of 599 × 599) and the output consists of 2 classes (DR and non-DR classes).

The automatic DR detection was based on a single fundus image per eye, macula-centered for both outcomes: any DR and mtmDR. After resizing, images obtained by the device were fed into the DL systems, without any other preprocessing.

### Statistical Analysis

Data were collected in MS Excel 2010 files (Microsoft Corporation). Statistical analyses were performed using SPSS 19.0 for Windows (SPSS Inc). Individuals’ characteristics and quantitative variables are presented in terms of mean and standard deviation. The chi-square test was used for the comparison of DR severity among races. The intergraders agreement was evaluated using the kappa (κ) statistic test, which ranges from 0 (no agreement or agreement that can be expected from random chance) to 1.00 (perfect agreement).^18^ The weighted κ was calculated based on International Classification of Diabetic Retinopathy classification subgroups. The 5% level of significance was used. Sensitivity, specificity, positive predictive value, and negative predictive value and their 95% confidence intervals (CIs) were calculated for the device outputs; for those calculations and also for the calculation of sample size, the populational prevalences were estimated according to available data in the literature.^19^ Artificial intelligence output was compared against human reading as the ground truth (see Tables S1 and S2). Diagnostic accuracy is reported according to the Standards for Reporting of Diagnostic Accuracy Studies.^20^

## Results

**Figure 2:**
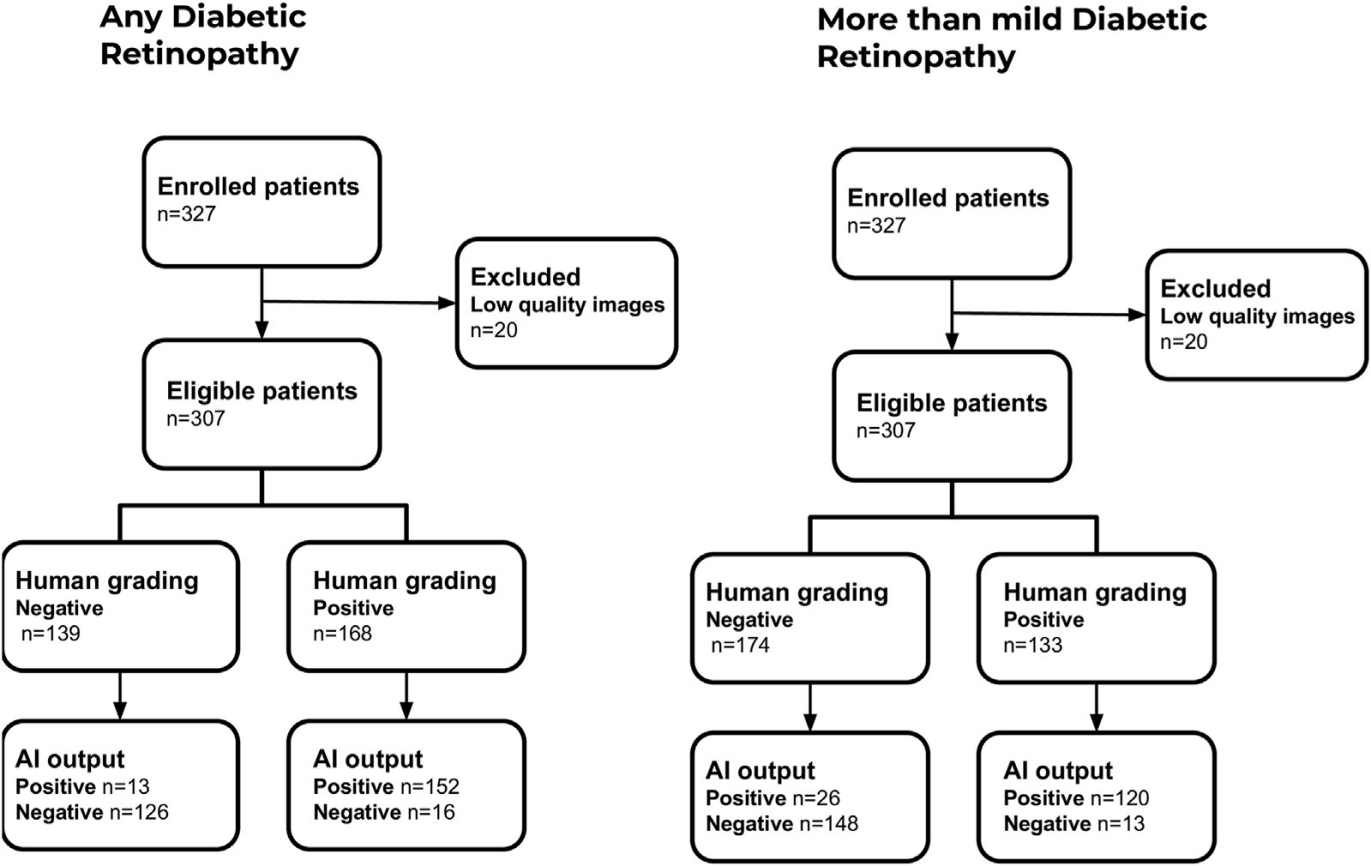
Waterfall diagram. Standards for Reporting of Diagnostic Accuracy Studies diagram for the algorithm. Eligible patients were analyzed by both the automatic systems and the human graders. Left: any diabetic retinopathy output. Right: more-than-mild diabetic retinopathy output. AI = artificial intelligence.

This study included retinal fundus photographs from 327 patients with a mean age of 57.0 (standard deviation, 16.8; range, 9–90) years and 45.3% male patients. Diabetes duration averaged 16.3 (± 9.7) years. Patients demographics and comorbidities are summarized in Table 1. Regarding DR classification, 44% of patients had no retinopathy, 26.47% had nonproliferative DR, and 29.31% had proliferative DR. The intergraders agreement regarding different categories of DR severity was very high (weighted κ, 0.895). After initial automatic assessment for quality, 20 patients (6.1%) who had their images graded by the human experts could not have them graded by the DL systems due to insufficient quality (Fig 2), resulting in a total of 307 individuals who had their images with sufficient quality for classification by both the specialist reader and the AI.

**Table 1:** Demographic Description and Clinical Characteristics of Enrolled Patients∗

	Value ± SD or (%)
Male sex	148 (45.26%)
Age, yrs	57.03 ± 16.82
Race†	
Mixed	133 (40.67%)
White	107 (32.72%)
Black	70 (21.42%)
Asian	10 (3.06%)
Indigenous	3 (0.92%)
Diabetes diagnosis, yrs	16.35 ± 9.69
Insulin use, yrs	11.67 ± 9.47
BMI, kg/m^2^	28.11 ± 5.37
Hypertension	221 (68%)

BMI = body mass index; SD = standard deviation.

∗ Values are presented as mean ± SD; percentages are indicated when adequate.

† Race distribution was not associated with diabetic retinopathy severity across the sample (*P* = 0.54).

The sensitivity and specificity, per the human grading standard, for the system to detect any DR was 90.48% (95% CI, 84.99%–94.46%) and 90.65% (95% CI, 84.54%–94.93%), respectively. Positive predictive value and negative predictive value for any DR were 73.49% (95% CI, 62.24%–82.34%) and 97.08% (95% CI, 95.41%–98.15%), respectively.

The sensitivity and specificity, per the human grading standard, for the system to detect mtmDR was 90.23% (95% CI, 83.87%–94.69%) and 85.06% (95% CI, 78.88%–90.00%), respectively. Positive predictive value and negative predictive value for mtmDR were 28.42% (95% CI, 21.71%–36.24%) and 99.25% (95% CI, 98.74%–99.55%), respectively.

**Figure 3:**
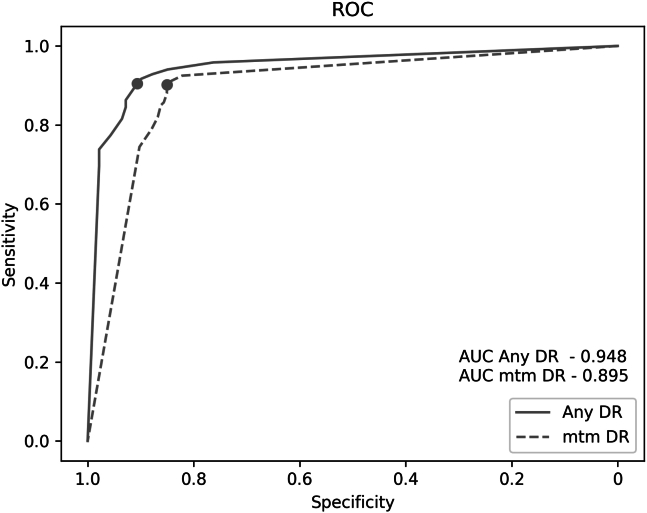
Receiver operating characteristic (ROC) curve of the artificial intelligence systems for detection of any diabetic retinopathy (DR) and more-than-mild DR (mtmDR). Area under the curve (AUC) for any DR: 0.948; AUC for mtmDR: 0.895.

The confusion matrices for both outcomes are displayed on Table 2. The area under the receiver operating characteristic curve was 0.948 for any DR and 0.895 for mtmDR (Fig 3).

**Table 2:** Confusion Matrices for the Outcomes: Any DR and mtmDR∗

	Expert Reading Positive	Expert Reading Negative	Total
Any DR predicted			
Positive	152 (TP)	13 (FP)	165
Negative	16 (FN)	126 (TN)	142
Total	168	139	307
mtmDR predicted			
Positive	120 (TP)	26 (FP)	146
Negative	13 (FN)	148 (TN)	161
Total	133	174	307

DR = diabetic retinopathy; FN = false negative; FP = false positive; mtmDR = more-than-mild diabetic retinopathy; TN = true negative; TP = true positive.

∗ Displayed results are valid for patients who underwent human and automatic analysis.

A qualitative post hoc analysis performed on incorrectly classified cases (false-positives and false-negatives, see Table 2) pointed to subtle losses of image quality in the majority of such cases: blurring, nonhomogenous fundus illumination, images with areas of low visibility, excessive brightness or overexposure, subtle losses of focus and/or image sharpness. Moreover, in some false negative cases, we have noticed the presence of microaneurysms or small hemorrhages; in selected false positive cases, we have observed the presence of drusen or pigment clumping in the macular area, tigroid or tessellated patterns of the fundus, or the persistence of myelinated fibers.

## Discussion

The present DR screening strategy with an automated detection system for the analysis of a single image per eye, obtained with a low-cost, portable retinal camera, achieved high sensitivity and specificity values for both outcomes: any DR and mtmDR. Of note, the achieved sensitivity values (90.48% vs. 90.23% for any DR and mtmDR, respectively) and specificity values (90.65% vs. 85.06% for any DR and mtmDR, respectively) were higher than the prespecified primary endpoint goals established for the first FDA approval of an automatic system for the detection of DR (sensitivity > 85% and specificity > 82.5%).^10^ The achieved values also compare well with those reported in the literature, both for strategies with traditional, tabletop retinal cameras and with portable devices.^5^^,^^21^, ^22^, ^23^, ^24^, ^25^

Limited access to ophthalmologists in many parts of the world poses a significant challenge in preventing avoidable blindness secondary to diabetes.^26^ In order to overcome challenges brought by the increasing numbers of diabetes cases worldwide, along with social and economic barriers to screening, telemedicine and AI have been established as cost-effective solutions.^5^^,^^17^ The employment of a smartphone-based device with an offline, embedded AI system could allow incremental coverage because of the portability and the potential of point-of-care results, even in areas without internet connection, further improving adherence rates and aiding in the prevention of visual loss.^9^^,^^27^

The portable device employed in the present study has previously been clinically validated for the detection of DR and tested in a real-world, high-burden setting.^27^^,^^28^ The adoption of low-cost, portable devices, such as the one reported herein, allows an easier screening process, especially in low-resource settings and hard-to-reach populations.^27^ Regarding the evolution of the imaging protocol for DR screening, we believe that the high performance achieved by automated evaluation of a single, macula-centered retinal image per eye, has the potential of making the process more friendly both for the operator and the patient; besides saving time, it is more likely to obtain good quality images in a single-field protocol than if multiple fields are required.

Other authors have recently assessed the performance of AI systems for DR evaluation using portable retinal cameras and yielding variable outcomes, including the detection of any DR, referable DR and sight-threatening DR; the main results of those studies are displayed in Table 3.^5^^,^^23^, ^24^, ^25^ The performance of our systems was comparable and often superior to the ones evaluated in those studies. Besides individual cameras’ and AI systems’ characteristics, such heterogeneity of performances may also be explained by different study designs; uneven sample sizes and variable data sets composition, especially the proportion of patients with and without the condition of interest, may also have influenced such variable results. Our sample was composed of 327 patients with diabetes, 26.47% with nonproliferative DR, and 29.31% with proliferative DR.

**Table 3:** Performance of AI Systems for the Detection of DR with Portable Retinal Cameras (Selected Studies)^5^^,^^5^, ^23^, ^24^, ^25^

Authors and Year	Population/Setting	Sample Size (Rate of DR)	Output	Sensitivity/Specificity (%)	Camera, AI System
Lupidi et al,^5^ 2023	Specialized Retina Clinic (Italy)	256 (50%)	Any DR	96.8/96.8	Optomed Aurora (Optomed, Oulu, Finland); Selena+
Ruan et al,^23^ 2022	Hospital-based (China)	315 (N/A)	Referable DR	88.2/40.7	Optomed Aurora (Optomed, Oulu, Finland); Phoebus
Rajalakshmi et al,^24^ 2018	Hospital-based (India)	296 (65%)	Any DR	95.8/80.2	Remidio (Remidio Innovative Solutions, India); EyeArt
			Sight-threatening DR	99.1/80.4	
Nunez do Rio et al,^25^ 2022	Community screening, rural and urban areas (India)	11 199 (3.8%∗)	Referable DR	72.08/85.65	Zeiss Visuscout (Carl Zeiss Meditec, Jena, Germany); VISUHEALTH-AI DR

AI = artificial intelligence; DR = diabetic retinopathy; N/A = not available.

∗ Referable DR.

The choice of different algorithm outputs among the mentioned studies also makes their comparison challenging, and this aspect is very relevant in terms of clinical application: the desired outcome has to suit local circumstances, the available health care workforce, financial constraints, and the intended goals of each program. The outcome of detecting mtmDR was chosen when the first autonomous system for DR evaluation was approved by the FDA.^10^ In our study, a good performance was achieved not only for mtmDR but also for the detection of any DR. Such “disease” versus “no disease” strategy has already been recognized as suitable as an assistive approach for reducing the burden on expert manual grading of retinal images, with a potentially favorable cost-to-benefit ratio regarding the number of human graders needed.^29^ Another reason for choosing the “any DR” outcome is related to the suggestion that a lower threshold for referrals is necessary when handheld cameras are employed for DR screening.^6^ Our research group has recently observed that eventual disagreements in the detection of microaneurysms, small hemorrhages, and intraretinal microvascular abnormalities may contribute to some discordance between portable devices and the traditional, tabletop retinal camera.^27^ Finally, separating patients with diabetes with DR from those without DR may also be a valuable tool if the risk for systemic events is to be evaluated.^12^

Ensuring high sensitivity and specificity rates is crucial for the success of a screening strategy. Although the systems reported herein attained a high performance, we also described some incorrectly classified cases. We believe that even subtle losses of image quality, corresponding to small changes in the system’s input, may have affected AI performance in a small fraction of the studied data set, occasionally resulting in false-negative or false-positive results. Based on the qualitative analysis of incorrectly classified cases, we believe future advances in automated detection of DR will include the improvement of automated quality assessment, to prevent images with suboptimal quality from being assessed by the algorithms for DR detection.^7^^,^^30^ In addition, DL systems for DR detection could be improved with model retraining and data augmentation of the training data set.

Besides a high performance, several other aspects must be implemented in order to guarantee that automatic systems are fully integrated into a clinical workflow and will actually lead to health improvements, and such factors depend on local circumstances such as regulatory issues, the legal framework, disease prevalence, availability of workforce, and economic constraints. It is important to recognize that, even if a DL system generates increased screenings and better referral adherence, access to subsequent specialty care might be unavailable within the current health care infrastructure.^31^ Further studies are needed for the evaluation of clinical outcomes and health economic metrics.^31^^,^^32^ Additionally, raising awareness about DR among patients and local health care workers is essential for the overall success of screening initiatives.^9^

We believe the main strength of this study lies in its design; besides being a multicenter study, it relied on a consistent and robust labeling performed by a reading center, and the data set was collected from an adequately balanced sample. Another important strength of the study is reporting the performance of automatic analysis evaluating a single image per eye for 2 different outcomes, representing different severity levels of DR; besides employing an automatic algorithm for the identification of retinal changes previously reported by our group,^17^ in the present study, we also evaluated a new algorithm that demonstrated high sensitivity and specificity for the detection of DR. The combination of both systems performed well in detecting mtmDR. Furthermore, we employed a previously validated device, not only regarding comparison to tabletop retinal cameras but also for being used in a real-world, high-burden setting.^27^^,^^28^

However, our study has limitations. The most important one is related to an analysis undertaken only on images with good quality, obtained by trained photographers, after pupil dilation, which precludes the extrapolation of our results in a real-world setting. Of note, some automatic systems of quality evaluation have been reported in this field.^7^^,^^30^ In addition, ours was not a real-world sample, because patients were recruited from reference centers, as opposed to patients attending screening programs. Finally, a good performance is a necessary but not sufficient condition for the success of the deployment of an AI-assisted screening program: results must be generalizable to different health care settings and among diverse demographic factors such as age, race, ethnicity, and socioeconomic status; clinical outcomes and economic analyses are also needed for real-world implementation.^31^ Nevertheless, we believe the present study adds value to the field of DR screening as a proof of concept, with further studies needed for the validation of the strategy with real-world data, such as the study authored by Bhaskaranand et al.^33^

In conclusion, with a growing diabetes epidemic and the global challenges faced by health systems to avoid blindness caused by diabetes, it is clear that portable and cost-effective devices and automatic systems will play a role in maximizing the outcomes of screening programs. The reported AI system, which attained a high performance for both outcomes of any DR and mtmDR, with the potential of being embedded in a portable device, working offline, and relying on just a single retinal image per eye, is a promising alternative for health systems overwhelmed by such a burden. Future studies are needed to evaluate the cost-effectiveness of such strategy and the feasibility of its deployment in the real world.
