# Supplementary material for: Automated Identification of Different Severity Levels of Diabetic Retinopathy Using a Handheld Fundus Camera and Single-Image Protocol
Source: Ophthalmol Sci. 2024 Feb 7;4(4):100481. doi: 10.1016/j.xops.2024.100481 (PMC11060947; doi:10.1016/j.xops.2024.100481)
Supplement: Table S1 [file mmc1.pdf]

Supplementary Material 1 –
Algorithmic performance for the detection of any diabetic retinopathy.

Threshold	Sensitivity	Specificity
**0**	100.00%	0.00%
**0.001**	95.83%	76.26%
**0.005**	94.05%	84.89%
**0.01**	92.86%	87.77%
**0.017**	91.67%	89.93%
**0.02**	**90.48%**	**90.65%**
**0.05**	86.31%	92.81%
**0.1**	84.52%	92.81%
**0.2**	81.55%	93.53%
**0.4**	77.38%	95.68%
**0.6**	73.81%	97.84%
**0.8**	69.64%	97.84%
**1**	0.00%	100.00%

The "threshold" column represents DRAS score values. Score values range from 0 (low probability of diabetic retinopathy) to 1 (high probability of diabetic retinopathy). The chosen threshold was 0.02.
