# Supplementary material for: Automated Identification of Different Severity Levels of Diabetic Retinopathy Using a Handheld Fundus Camera and Single-Image Protocol
Source: Ophthalmol Sci. 2024 Feb 7;4(4):100481. doi: 10.1016/j.xops.2024.100481 (PMC11060947; doi:10.1016/j.xops.2024.100481)
Supplement: Table S2 [file mmc2.pdf]

Supplementary Material 2 –
Algorithmic performance for the detection of more than mild diabetic retinopathy.

Threshold	Sensitivity	Specificity
**0**	100.00%	0.00%
**0.001**	92.48%	82.18%
**0.005**	90.98%	84.48%
**0.01**	**90.23%**	**85.06%**
**0.017**	88.72%	85.06%
**0.02**	87.97%	85.06%
**0.05**	85.71%	85.63%
**0.1**	84.96%	86.21%
**0.2**	81.95%	86.78%
**0.4**	78.95%	87.93%
**0.6**	76.69%	89.08%
**0.8**	74.44%	90.23%
**1**	0.00%	100.00%

The “threshold” column represents the combined RAS+DRAS score values. Score values range from 0 (low probability of more than mild diabetic retinopathy) to 1 (high probability of more than mild diabetic retinopathy). The chosen threshold was 0.01.
