# Supplementary material for: Automated Identification of Different Severity Levels of Diabetic Retinopathy Using a Handheld Fundus Camera and Single-Image Protocol
Source: Ophthalmol Sci. 2024 Feb 7;4(4):100481. doi: 10.1016/j.xops.2024.100481 (PMC11060947; doi:10.1016/j.xops.2024.100481)
Supplement: Appendix S1 [file mmc3.pdf]

Supplementary Material 3

Computer Science Selected Literature Related to the Models presented in this research

Xception

Chollet, François. "Xception: Deep learning with depthwise separable convolutions." *Proceedings of the IEEE conference on computer vision and pattern recognition*. 2017.

EfficientNet v2s

Tan, Mingxing, and Quoc Le. "Efficientnetv2: Smaller models and faster training." *International conference on machine learning*. PMLR, 2021.

TensorFlow

Abadi, Martín, et al. "Tensorflow: Large-scale machine learning on heterogeneous distributed systems." *arXiv preprint arXiv:1603.04467* (2016).

Keras

Keras: Deep Learning for humans

GitHub - keras-team/keras: Deep Learning for humans

Chollet, F., & Others. (2015). Keras. Retrieved from https://keras.io
